# Suspected Fat Embolism Syndrome in the Setting of Ballistic Long Bone Fractures: A Case Report

**DOI:** 10.5811/cpcem.35398

**Published:** 2025-09-05

**Authors:** Irfan Husain, Danielle Andrews

**Affiliations:** Emory University School of Medicine, Department of Emergency Medicine, Atlanta, Georgia

**Keywords:** case report, fat embolism syndrome, trauma, orthopedics, fracture

## Abstract

**Introduction:**

Fat embolism syndrome (FES) is a rare, life-threatening condition most seen in traumatic orthopedic injuries, especially long bone fractures. Classically, FES presents with hypoxemia, neurological abnormalities, or a petechial rash; however, clinical findings can extend beyond this classic triad. Since FES is a clinical diagnosis, emergency physicians must recognize both classic and subtle presentations.

**Case Report:**

A 22-year-old female presented as a transfer from an outside hospital for multiple long bone fractures secondary to gunshot wounds. Upon arrival, she was found to be hypoxic, despite no signs of thoracic injury on exam or initial imaging. Her presentation, laboratory findings, and repeat imaging were consistent with FES. She was given supportive care through supplemental oxygen and close monitoring. She improved with supportive care and was discharged home in stable condition.

**Conclusion:**

Although there is no definitive treatment for fat embolism syndrome, prompt recognition of the various clinical findings associated with FES by emergency physicians can expedite supportive care, allow prompt admission to a critical care unit, and aid with monitoring for potential deterioration.

## INTRODUCTION

Fat emboli occur when fat globules enter the circulation; however, the presence of these globules may or may not cause signs or symptoms. Fat emboli only become clinically significant when they progress to a rarer condition known as fat embolism syndrome (FES). Although not well defined, FES refers to the various clinical findings of end organ dysfunction that result from the dissemination of fat emboli into circulation.^1^ It is thought that as the fat emboli enter the microcirculation and disrupt the capillary bed, they can cause a systemic inflammatory reaction affecting multiple organ systems. Fat embolism syndrome can be challenging to diagnose as signs and symptoms can be non-specific, and there is no gold standard test. Thus, FES is a clinical diagnosis and a diagnosis of exclusion.^1^–^3^ The incidence of FES is dependent on the criteria used for diagnosis and has been estimated to be as high as 11% and as low as 1%.^2^

We report a case of a young female who was transferred from an outside hospital for multiple ballistic fractures to her left leg secondary to a high-powered rifle. Upon arrival to the trauma center nearly 12 hours from onset of injury, the patient was found to be hypoxic to mid 80% on room air. We review the patient’s clinical course and the findings that led to a diagnosis of FES. The case report highlights signs and symptoms, different diagnostic criteria, laboratory and imaging findings, and the management of FES. This case is unique in that it presents chest computed tomography (CT) from before and after the suspected development of FES, highlighting its characteristic features on imaging.

## CASE REPORT

A 22-year-old female patient with no prior medical history presented to the emergency department (ED) of a Level I trauma center as a transfer. She had been shot multiple times in the left lower extremity with a high-powered rifle. She was taken to a nearby hospital for stabilization. Labs, plain films, CT of the chest, abdomen and pelvis with intravenous (IV) contrast, and a CT angiogram of the lower extremities were completed. Plain films of the left lower extremity revealed a fracture of the lateral femoral condyle and non-displaced distal tibia and fibula fractures. The CT angiogram showed a lack of opacification of the left posterior tibial artery at the level of the ankle, with normal distal opacification and preservation of surrounding arteries of the foot. The radiology read suggested this could represent vasospasm, although vessel injury could not be excluded. The CT of the chest, abdomen and pelvis with IV contrast revealed no evidence of intrathoracic or intra-abdominal injuries. She was then transferred to the Level I trauma center for higher level of care.

The patient presented to our ED approximately 12 hours from the time of the incident. Her primary survey was intact, and she had a Glasgow Coma Score of 15. Her vitals were significant for an oxygen saturation in the mid 80% on room air and a heart rate in the low 100 beats per minute. Her remaining vitals were within normal limits. She was subsequently placed on four liters of oxygen via nasal cannula, which improved her oxygen saturation to within normal limits. There was no mention of hypoxia or an oxygen requirement in the outside hospital records. Her secondary survey was significant for large ballistic wounds to the anterolateral lower leg, the posteromedial lower leg, and the lateral knee. She had a palpable dorsalis pedis in her left foot with an ankle brachial index greater than 1. There were no signs of trauma to her torso.

**Image 1:**
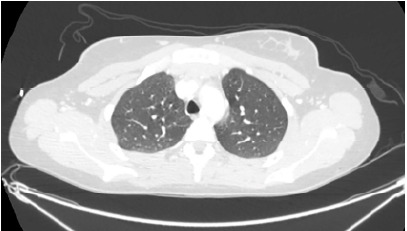
Outside hospital computed tomography chest showing no acute abnormalities.

**Image 2:**
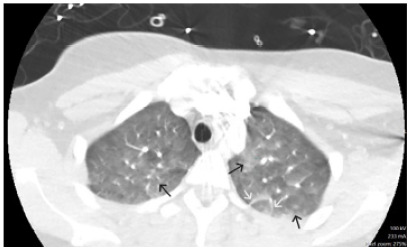
Repeat computed tomography chest post hypoxia. Black arrows indicate ground-glass opacities. White arrows indicate smooth interlobular septal thickening.

The trauma surgeons reviewed the outside imaging and determined no arterial vascular injury. Orthopedic surgery was consulted, and she was placed in a long leg splint with anticipation for an open reduction internal fixation surgery. During her workup, the patient began to complain of increasing shortness of breath. A repeat chest radiograph done upon arrival showed no acute findings. She became increasingly hypoxic and tachypneic and eventually required high-flow nasal canula (HFNC). We began to suspect FES, given the hypoxia in the setting of a delayed presentation of long bone fractures. To further evaluate for FES, and to rule out a pulmonary embolism, a CT angiogram of the chest was performed. The CT showed scattered bilateral upper lobe peribronchovascular ground-glass opacities with trace nodular component, as well as smooth interlobular septal thickening concerning for pulmonary edema or FES. Image 1 shows a cross-sectional view of the unremarkable CT chest from the outside hospital. Image 2 shows the CT chest completed after the patient became hypoxic and demonstrates common findings seen in FES, such as ground-glass opacities and smooth interlobular septal thickening.

The patient’s B-type natriuretic peptide was within normal limits at 14 picograms per milliliter (pg/mL) (reference range: less than 79 pg/mL), and she showed no signs of volume overload on exam. A reassessment of her physical exam just prior to admission showed a new finding of non-blanching petechiae to her left lateral upper thigh. The patient was ultimately admitted to trauma surgery.

*CPC-EM Capsule*

What do we already know about this clinical entity?

*Fat embolism syndrome is a rare complication of orthopedic injuries. It classically presents with hypoxemia, neurological changes, or petechial rash and management is supportive*.

What makes this presentation of disease reportable?

*This case provides pre- and post-hypoxia computed tomography imaging, highlighting common radiographic changes consistent with fat embolism syndrome*.

What is the major learning point?

*Delayed hypoxia in orthopedic trauma should prompt consideration of fat embolism syndrome, which ultimately is a clinical diagnosis*.

How might this improve emergency medicine practice?

*Improves early recognition of fat embolism syndrome by highlighting key clinical, laboratory, and imaging findings*.

The echocardiogram completed after admission showed normal systolic function with mild-to-moderate pulmonary hypertension and mild tricuspid regurgitation. During her 11-day hospital stay, the patient developed anemia and thrombocytopenia, both of which can develop in patients with FES. At the outside hospital, her initial hemoglobin was 12.8 grams per deciliter (gm/dL) (11.3–15.0 gm/dL), and her platelet count was 226,000/microliter (mcL) (150,000–450,000/mcL). Her hemoglobin dropped to 7.3 gm/dL (day five) and platelets to 98,000/mcL (day three), both improving without any blood products by time of discharge. During her stay the patient underwent operative fixation of her fractures and multiple irrigation and debridements with minimal blood loss documented. The inpatient team initially managed her hypoxia with HFNC but successfully weaned her to low-flow nasal cannula by day three, and eventually to room air before discharge.

## DISCUSSION

Fat embolism syndrome can result from both traumatic and non-traumatic causes; non-traumatic causes are much rarer. Non-traumatic causes can include fatty liver, pancreatitis, bone marrow transplantation, and liposuction. Traumatic causes can include long bone fractures, pelvic fractures, intramedullary nailing, knee and pelvic arthroplasty, severe burns, and crush injuries.^2^ Fat embolism syndrome typically occurs anywhere from 12–72 hours after injury.^1^ Our case highlights a patient with suspected FES secondary to multiple long bone fractures. The presence of multiple long bone fractures further increased her risk for developing FES. A 2008 study of *International Classification of Diseases*, *9^th^ Rev*, codes from 1979–2005 in the National Hospital Discharge Survey found that the relative risk of FES in patients with multiple fractures (including the femur) compared with an isolated femur fracture was 2.35.^4^

Fat embolism syndrome can be difficult to identify given the wide array of clinical features and the lack of a gold standard test. It remains a clinical diagnosis that requires evaluation of a patient’s signs and symptoms in combination with supporting lab and imaging findings. Fat embolism syndrome classically presents with respiratory, neurologic and skin abnormalities.^5^ Severe cases of FES can lead to heart failure, acute respiratory distress syndrome, cerebral edema, and shock.^1^ It is estimated that 10–44% of patients will require non-invasive or invasive ventilation.^5^

Currently, there are no validated or widely accepted diagnostic criteria for FES. However, over the years, several authors have proposed frameworks to aid in its identification. These include the Gurd and Wilson criteria, the Schonfeld Fat Embolism Index, and the Lindeque criteria, which emphasize common signs, symptoms, lab abnormalities, and imaging findings associated with FES.^6^–^8^ The table presents all three diagnostic criteria, outlining their respective features and scoring systems.

The Lindeque criteria, which use respiratory symptoms and blood gas findings alone, are not as well accepted as the other two criteria.^8^ Ultimately, concern for FES should not rely strictly on diagnostic criteria but rather the emergency physician’s clinical suspicion and ability to rule out other potential causes.

We believe the clinical course supported a diagnosis of FES for our patient. Nearly 12 hours from the onset of injury she developed acute onset tachypnea and hypoxia requiring eventual HFNC. There was no blunt trauma or signs of injury to the chest. During her initial evaluation at the outside hospital, she had an unremarkable chest CT; however, when the CT was repeated nearly 12 hours later, she had developed the classic CT findings associated with FES. The most common pattern described on chest CT is patchy, ground- glass opacities that are often associated with smooth interlobular septal thickening.^3^,^9^ The patient also had an echocardiogram that showed evidence of pulmonary hypertension and tricuspid regurgitation. These findings have been previously documented in cases of FES.^10^,^11^ A point-of-care echocardiogram during the initial assessment may have been beneficial, possibly revealing small, floating, hyperechoic particles representing fat emboli in the inferior vena cava.^12^ The patient later developed supporting skin findings including petechia to the upper portion of her leg and supporting lab findings of an acute drop in her hemoglobin and platelets.

The mainstay treatment for FES is supportive care. This includes treating hypoxemia with supplemental oxygen, managing hypovolemia with fluid resuscitation or vasopressors, and addressing severe anemia with packed red blood cells. Extracorporeal membrane oxygenation should be considered for refractory hemodynamic instability. Close monitoring of neurologic status with frequent neuro checks is essential. Admission to the intensive care unit is strongly recommended for optimal management.^1^ For our patient, the inpatient team provided supportive care through supplemental oxygen via HFNC. Eventually the patient was weaned off oxygen and discharged in stable condition. Pharmacologic therapies have been studied, but none widely accepted. The most extensively studied is corticosteroids for purposes of FES prevention. A 2009 meta-analysis of seven, low-quality, randomized controlled trials showed a reduction in the risk for FES and resulting hypoxia in long bone fractures, but no mortality benefits.^13^ Therefore, the role of corticosteroids remains controversial.

## CONCLUSION

Fat embolism syndrome is a rare condition that results from the release of fat emboli into circulation and can lead to various types of end-organ dysfunction. Fat embolism syndrome is commonly associated with traumatic orthopedic injuries and classically results in hypoxemia, neurologic dysfunction, or a petechial rash, among other symptoms. Fat embolism syndrome remains a clinical diagnosis, determined by presentation, laboratory findings, and imaging. The mortality rate is estimated to be nearly 12%, and the mainstay treatment remains supportive care.^14^

## Figures and Tables

**Table:** Criteria for diagnosis of fat embolism syndrome.

Criteria	Features
Gurd and Wilson FES = 1 major + 4 minor + fat macroglobulinemia	Major Criteria
	Respiratory insufficiency
	Cerebral involvement
	Petechial rash
	Minor Criteria
	Pyrexia
	Tachycardia
	Retinal changes
	Jaundice
	Renal changes
	Anemia
	Thrombocytopenia
	Elevated erythrocyte sedimentation rate
	Fat macroglobulinemia
Schonfeld Fat Embolism Index FES = Score > 5	Petechial rash (5 points)
	Diffuse alveolar infiltrates (4 points)
	Hypoxemia - PaO_2_ < 70 mmHg (3 points)
	Confusion (1 point)
	Fever ≥ 38 °C (1 point)
	Heart rate ≥ 120/min (1 point)
	Respiratory rate ≥ 30/min (1 point)
Lindeque FES = femur fracture ± tibia fracture + 1 feature	PaO_2_ < 60 mmHg
	PaCO_2_ > 55 mmHg
	Respiratory rate > 35/min, despite sedation
	Dyspnea, tachycardia, anxiety

*FES*, fat embolism syndrome*; PaO_2_*, partial pressure of oxygen in arterial blood; *mmHg*, millimeters of mercury; *PaCO_2_*, partial pressure of carbon dioxide in arterial blood.
